# A GIS-based 3D slope stability analysis method based on the assumed normal stress on the slip surface

**DOI:** 10.1038/s41598-020-61301-x

**Published:** 2020-03-10

**Authors:** Guo Yu, Mowen Xie, Jun Liang, Asim Farooq, Edward J. Williams

**Affiliations:** 1 School of Civil and Resource Engineering, University of Science & Technology Beijing, Beijing, 100083 China; 2 Geological Bureau of Shenzhen, Shenzhen, 518023 China; 3 Department of Civil Engineering, CECOS University of IT & Emerging Sciences, Peshawar, Pakistan; 4 Department of Science and Engineering University of Michigan, Dearborn, United States of America

**Keywords:** Natural hazards, Natural hazards, Computational science, Computational science

## Abstract

The study proposes a geographic information systems (GIS)-based slope stability analysis method assuming a normal stress distribution acting on the slip surface. Compared with traditional methods, the three-dimensional (3D) safety factor acquired through this method will more closely approximate the actual value. First, a 3D slope stability analysis model is developed using grid column units, and the spatial expression of calculation parameters based on the grid column is given by the spatial analysis capability of GIS. Then, four equilibrium equations are derived under the limit equilibrium condition. The normal stress distribution acting on the slip surface is analyzed to construct a reasonable normal stress distribution approximation function. The 3D safety factor is obtained through the approximation function and the Mohr-Coulomb strength criterion. Moreover, we develop a GIS-based extension module which combines the grid-based data with the 3D slope stability analysis model. The accuracy and feasibility of the module are verified by three typical cases.

## Introduction

The limit equilibrium approach is commonly used in engineering applications for evaluating slope stability^1–3^. As algorithms have advanced in sophistication, scholars have increasingly and more productively studied the stability of three-dimensional (3D) slopes. Canonically, the sliding body is divided into vertical columns, and under the assumption of inter-column forces, different 3D limit equilibrium methods are developed for the 3D slope safety factor by different force and moment balance conditions. For example, Hovland^4^, Hungr^5^, and Boutrup^6^ extended the two-dimensional (2D) model developed by Fellenius, Bishop and Janbu to 3D, but such methods need to assume inter-column forces, and their iterative calculations may not converge^7^.

Therefore, scholars proposed to improve the limit equilibrium methods by assuming the normal stress distribution acting on the slip surface. Assuming a normal stress distribution, Bell^8^ and Zhu and Lee^9^ proposed that the normal stress distribution acting on the slip surface be considered a function with two parameters that need to be determined. Zhu^10,11^ and Zheng^12^ proposed that the normal stress distribution acting on the slip surface was composed of an initial function and a correction function, and the correction function was assumed to be a linear interpolation function with two parameters to be determined. This method does not need to assume the inter-column forces, and compensates for flaws in the integration of calculations in conventional methods; however, these assumed functions cannot effectively simulate the actual normal stress distribution acting on the slip surface, which leads to inaccurate computational results^7^.

At the same time, although many commercial software applications using the 3D limit equilibrium method (3D-LEM) have also been developed, the data formats for these applications are different, making them inconvenient for engineers to use, which is not conducive to secondary development. Therefore, geographic information systems (GIS) technology having powerful spatial data processing capability was introduced into the 3D slope stability analysis^13^. GIS can provide a uniform platform and data structure for spatial computing problems. All the data related to slope can be expressed in a raster dataset based on the grid column unit, which makes data processing and calculation more convenient. Therefore, the 3D slope stability analysis model can be established based on grid column units.

Studies have investigated the combination of GIS and mechanical model. The Current Study^14–16^ extended the 2D Hovland model, Bishop Model and Janbu model to 3D models; we developed 3D slope stability analysis software based on GIS, called 3Dslope. Mergili^17^ combined GRASS GIS and the 3D Hovland model to implement a 3D Slope stability model which can accommodate both shallow and deep-seated slope failures. The above mechanical models were combined mainly with GIS by assuming inter-column forces. However, the establishment of 3D-LEM based on the assumed normal stress distribution acting on the slip surface in GIS has not been studied by scholars, and its algorithm is very different from the above mechanical models. The key to this method is in its assumption about the normal stress distribution acting on the slip surface based on GIS. The accuracy of measurements using the method is proportional to the reasonableness of this assumption.

This study proposes a method for analyzing stability of the 3D slope based on GIS which assumes normal stress distribution acting on the slip surface. The composition of the normal distribution of stress is evaluated and we recommend an approximation method to represent the actual normal distribution of stress acting on the slip surface. Compared with traditional methods, the resulting value calculated by using the approximation function is closer to the actual value. To make the calculation more convenient, a GIS-based extension module is developed to evaluate slope stability. This module overcomes the problem of inconsistent data format and difficulty in secondary development. The accuracy and feasibility of this method are verified by three cases.

## Implementation of 3D limit equilibrium method in GIS

### 3D slope model based on GIS

**Figure 1:**
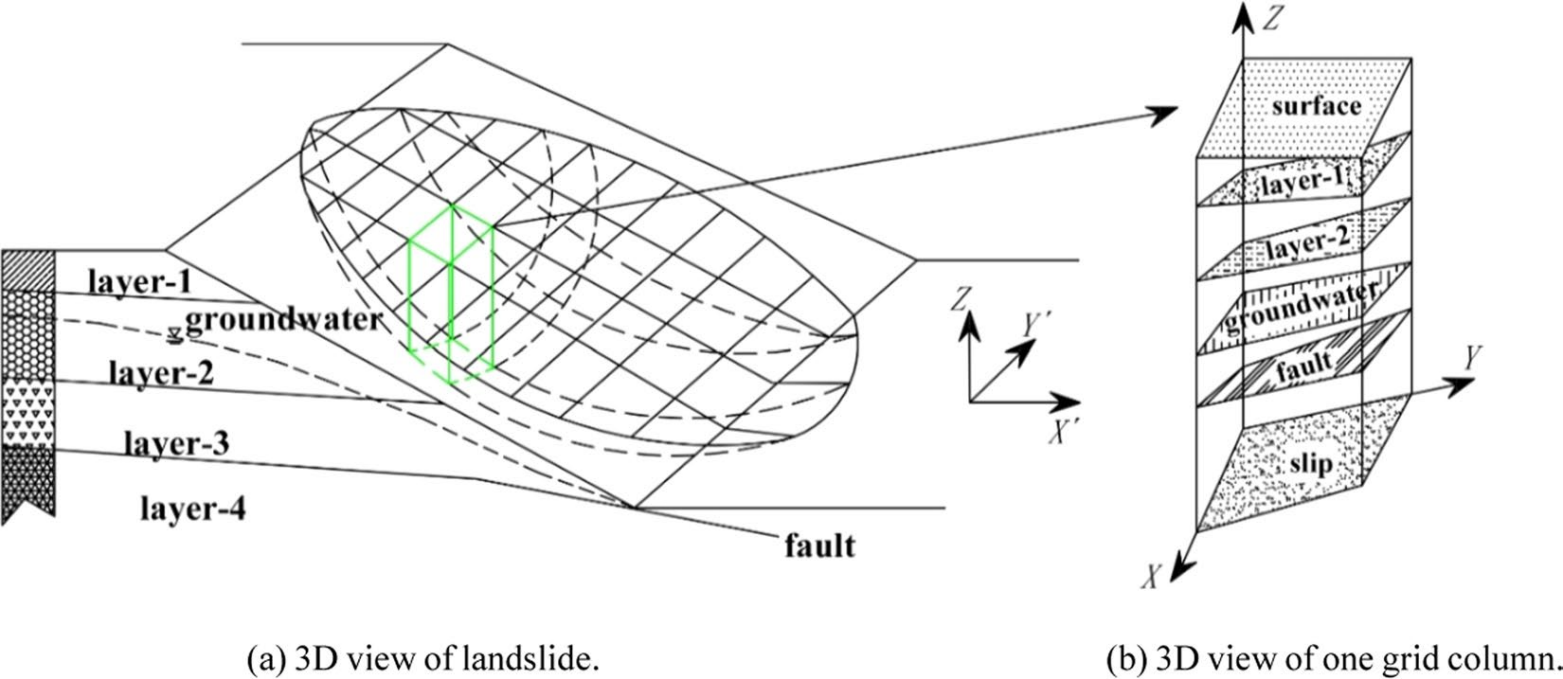
3D view of landslide and one grid column. (**a**) 3D view of landslide. (**b**) 3D view of one grid column.

Figure 1(a) Shows a 3D sliding body divided into grid columns. Each column contains all the data related to the slope, such as surface, strata, groundwater, fault, slip, etc., as shown in Fig. 1(b). With GIS, the parameters of each column for solving the 3D safety factor can be obtained, such as elevation, inclination, slope angle, etc.

**Figure 2:**
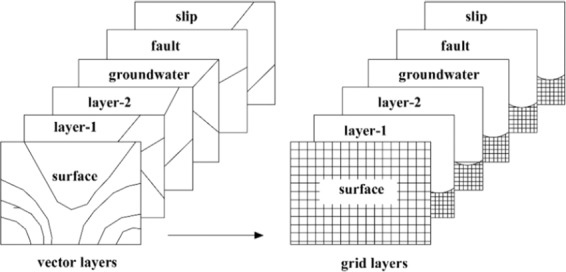
GIS layers for slope stability analysis.

Without GIS it would be a tedious and time-consuming process to measure the 3D safety factor based on a column model. However, in the GIS system, the data related to the slope can be abstracted into a vector layer by using the functions of GIS spatial analysis, and the vector layer can be converted into a grid layer, as shown in Fig. 2. The grid size (cell size) can be set appropriately to achieve the requisite precision. All grid layers in a column relating to the slope are combined to form a segment of the grid column. Based on the grid column model, the calculation of the 3D safety factor will be routine and canonical.

**Figure 3:**
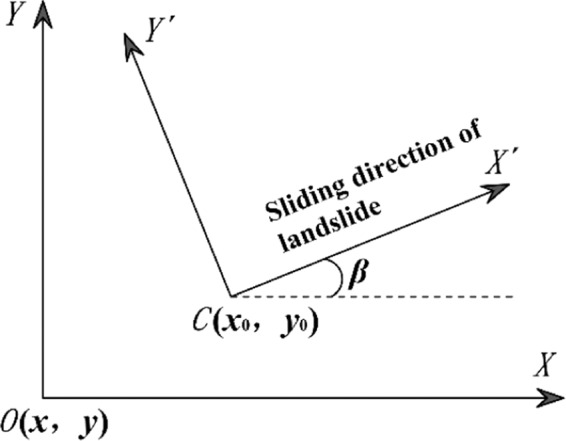
Coordinate system conversion.

To facilitate subsequent calculations, the *XOY* coordinate system was converted to an *X*′*CY*′ coordinate system. The *X*′-axis direction was defined as the sliding direction of the landslide. The right-hand rule determined the positive directions of the *Y*′- and *Z*-axes. In addition, point *O*, i.e., the origin of the *XOY* coordinate system, was translated to point *C* in the *X*′*CY*′ coordinate system, as shown in Fig. 3. The transformation of the coordinates can be expressed as follows:

1 $$\{\begin{array}{c}x{\prime} \\ y{\prime} \end{array}\}=(\begin{array}{c}\cos (90-\beta )\,\sin (90-\beta )\\ -\,\sin (90-\beta )\,\cos (90-\beta )\end{array})\{\begin{array}{c}x-{x}_{0}\\ y-{y}_{0}\end{array}\}$$

where *β* is the sliding direction of the landslide, defined as the main dip direction of the landslide. The main dip direction of the landslide is the most frequent value of the dip directions of all grid columns. *x*′ and *y*′ are the coordinate values of the center of the bottom of each grid column in the *X*′*CY*′ coordinate system. *x* and *y* are the coordinate values of the center of the bottom of each grid column in the *XOY* coordinate system. *x*_0_ and *y*_0_ are the coordinate values of point *C* in the *XOY* coordinate system.

### Force analysis of one grid column

**Figure 4:**
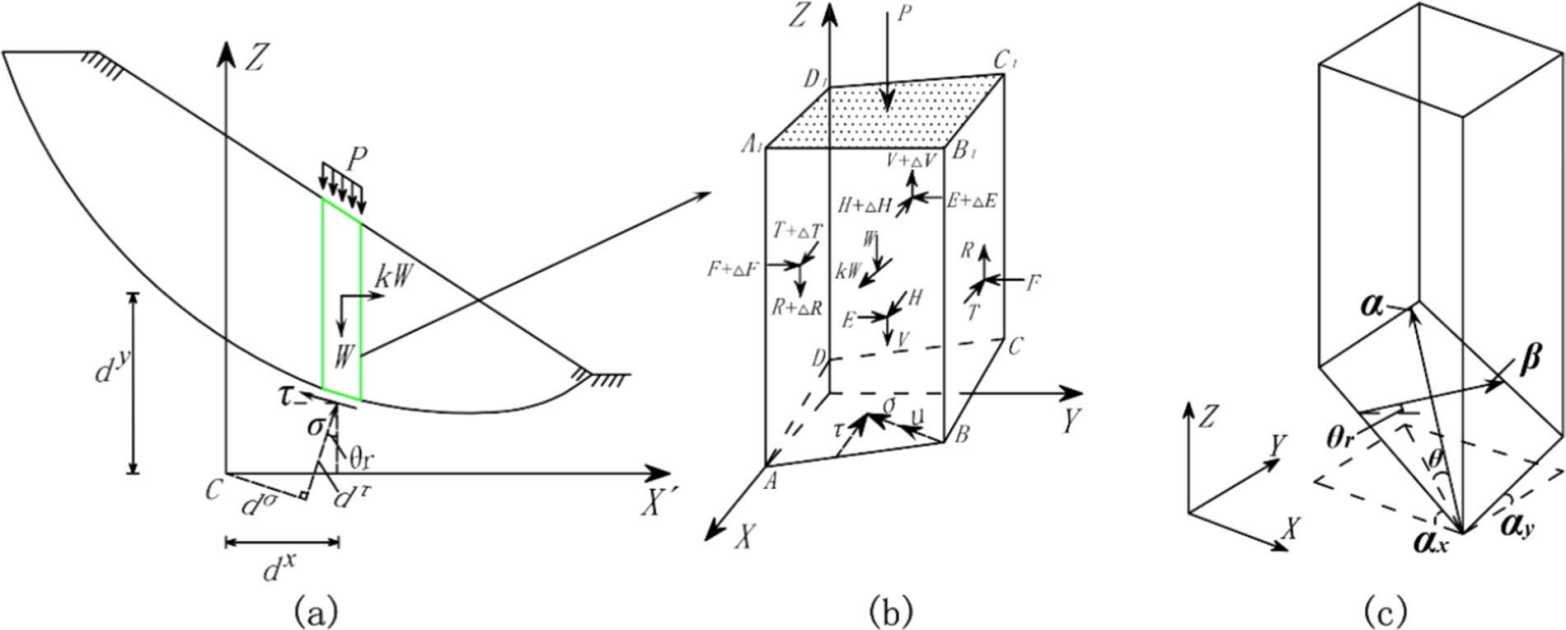
Force analysis of one grid column and the 3D spatial relationship. (**a,b**) Show the force analysis of one grid column in the 2D and 3D views of the slope, respectively, and (**c**) shows the spatial relationship in the 3D view of the slope.

Figure 4 shows the force analysis of one grid column and its 3D spatial relationship. We can specify the forces acting on each grid column as follows:

1. The weight of one grid column is *W*; the direction is the *Z*-axis, and the weight acts at the centroid of the grid column.
2. The resultant horizontal seismic force is *kW*, where *k* is the “seismic coefficient”; the direction of *kW* is the *X*′-axis, and the resultant horizontal force acts at the centroid of the grid column.
3. The external loads on the ground surface are represented by *P*; the direction of *P* is the *Z*-axis, and these external loads act at the center of the top of the grid column.
4. The normal and shear stresses acting on the slip surface are represented by *σ* and *τ*, respectively. Normal stress is directed perpendicular to the slip surface; shear stress is directed in the sliding direction of the landslide. The normal and shear stresses work at the base of the grid column’s centroid.
5. The pore water pressure on the slip surface is *u*.
6. The moment arm of *P* and *W* around the *Y*′-axis is *d*^*x*^; the moment arm of *kW* around the *X*′-axis is *d*^*y*^; the moment arm of *σ* around the *Y*′-axis is *d*^*σ*^; and the moment arm of *τ* around the *Y*′-axis is *d*^*τ*^, as shown in Fig. 3(a).
7. The horizontal tangential forces on the vertical face at *y* = 0 and vertical face at *y* = Δ*y* (Δ*y* represents the size of the grid column along *Y*-axes) are *T* and *T* + Δ*T*, respectively; the vertical tangential forces on the vertical face at *y* = 0 and vertical face at *y* = Δ*y* are *R* and *R* + Δ*R*, respectively; the normal forces on the vertical face at *y* = 0 and vertical face at *y* = Δ*y* are *F* and *F* + Δ*F*, respectively; the horizontal tangential forces on the vertical face at *x* = 0 and vertical face at *x* = Δ*x* are *E* and *E* + Δ*E*, respectively; the vertical tangential forces on the vertical face at *x* = 0 and vertical face at *x* = Δ*x* are *V* and *V* + Δ*V*, respectively; and the normal forces on the vertical face at *x* = 0 and vertical face at *x* = Δ*x* are *H* and *H* + Δ*H*, respectively, as shown in Fig. 3(b).
8. *θ* is the dip of the grid column on the slip surface; α is the dip direction of the grid column on the slip surface; *θ*_*r*_ is the apparent dip of the main inclination direction of the landslide; *a*_*x*_ is the apparent dip of the *X*-axis; and *a*_*y*_ is the apparent dip of the *Y*-axis, as shown in Fig. 3(c).

### 3D limit equilibrium equations based on GIS

As with many other limit equilibrium methods, the safety factors for each grid column on its respective slip surfaces are assumed to be equal. The 3D safety factor is derived from the Mohr-Coulomb strength criterion as follows:

2 $$S{F}_{3D}=\frac{c{\prime} +\sigma \,\tan \varphi {\prime} }{\tau }$$

where *SF*_3D_ is the 3D safety factor, *φ*′ is the effective friction angle, and *c*′ is the effective cohesion.

As shown in Fig. 4, for the entire 3D sliding body, the total intercolumn force in the *X*′, *Y*′, and *Z* directions and the total moment around the *Y*′-axis are zero. Therefore, the force equilibrium equations in the *X*′, *Y*′ and *Z* directions and moment equilibrium equation around the *Y*′-axis become

3 $$X{\prime} =\sum _{I}\sum _{J}\{A\tau \,\cos \,{\theta }_{r}-A(\sigma +u)\sin \,\theta \,\cos (\alpha -\beta )-kW\}=0$$

4 $$Y{\prime} =\sum _{I}\sum _{J}A(\sigma +u)\sin \,\theta \,\sin (\alpha -\beta )=0$$

5 $$Z=\sum _{I}\sum _{J}(A\tau \,\sin \,\theta r+A(\sigma +u)\cos \,\theta -W-P)=0$$

6 $$M=\sum _{I}\sum _{J}\{(W+P){d}^{x}+kW{d}^{y}-(\sigma +u){d}^{\sigma }-A\tau \,\cos (\alpha -\beta ){d}^{\tau }\}=0$$

where

7 $${d}^{x}=x{\prime} ,{d}^{y}=0.5\,h+z$$

8 $${d}^{\sigma }=(x{\prime} -z\,\tan \,\theta r)\cos \,\theta r$$

9 $${d}^{\tau }=(x{\prime} -z\,\tan \,\theta r)\sin \,\theta r+\frac{z}{\cos \,\theta r}$$

10 $$W=cellsiz{e}^{2}\mathop{\sum }\limits_{i=1}^{n}hi\gamma i,P=cellsiz{e}^{2}p,u=\frac{D}{\cos \,\theta }$$

where *I* and *J* represent the numbers of rows and columns of the grid, respectively; *z* represents the coordinate values of the center of the bottom of each grid column; *h* is the height of the grid column; *h*_*i*_ is the height of each stratum; and *γ*_*i*_ is the natural unit weight of each stratum; *D* is the distance from the center of the bottom of the grid column to the water surface.

From Fig. 3(c), the apparent dips of the *X*-axis and *Y*-axis are

11 $$\tan \,{a}_{x}=\,\cos \,\alpha \,\tan \,\theta ,\,\tan \,{a}_{y}=\,\sin \,\alpha \,\tan \,\theta $$

The slip surface area of one grid column is calculated by

12 $$A=cellsiz{e}^{2}\left[\frac{\sqrt{(1-{\sin }^{2}{a}_{x}{\sin }^{2}{a}_{y})}}{\cos \,{a}_{x}\,\cos \,{a}_{y}}\right]$$

The apparent dip of the landslide’s main inclination direction is determined by:

13 $$\tan \,{\theta }_{\gamma }=\,\tan \,\theta |\cos (\alpha -\beta )|$$

When combining Eqs. (2–6), the equation set is established to solve the 3D safety factor. Since the equation set includes (*I* × *J* + 1) unknowns, i.e., *SF*_3D_, and (*I* × *J*) normal stresses *σ*, the equation set cannot be solved. If the normal stress distribution on the slip surface is known, this equation set can be solved.

## Analysis of the Composition of Normal Stress Distribution *σ*

As shown in Fig. 4(b), the force balance equation for the *X*′-, *Y*′- and *Z*-axes can be obtained according to the limit equilibrium condition of a single grid column and without any assumption on force as follows:

14 $$X{\prime} =A\tau \,\cos \,{\theta }_{r}-A(\sigma +u)\sin \,\theta \,\cos (\alpha -\beta )-kW+\Delta T-\Delta H=0$$

15 $$Y{\prime} =A(\sigma +u)\sin \,\theta \,\sin (\alpha -\beta )+\Delta F-\Delta E=0$$

16 $$Z=A\tau \,\sin \,\theta r+A(\sigma +u)\cos \,\theta -W-P+\Delta V-\Delta R=0$$

Subtracting *τ* from Eqs. (14–16) allows the expression of the normal stress distribution function on the slip surface to be obtained as

17 $$\begin{array}{rcl}\sigma  & = & \frac{(W+P)\cos \,\theta r(\sin \,\beta +\,\cos \,\beta )-kW\,\sin \,\theta r}{A\,\cos \,\theta \,\cos \,\theta r(\sin \,\beta +\,\cos \,\beta )+A\,\sin \,\theta \,\sin \,\theta r(\sin \,\alpha +\,\cos \,\alpha )}-u\\  &  & +\frac{(\Delta V-\Delta R)\cos \,\theta r(\sin \,\beta +\,\cos \,\beta )-(\Delta T-\Delta H+\Delta F-\Delta E)\sin \,\theta r}{A\,\cos \,\theta \,\cos \,\theta r(\sin \,\beta +\,\cos \,\beta )+A\,\sin \,\theta \,\sin \,\theta r(\sin \,\alpha +\,\cos \,\alpha )}\end{array}$$

Using Eq. (17), we conclude that the normal stress distribution *σ* acting on the slip surface can be divided into two parts. The first part comes from the weight *W*, external loads *P*, the pore water pressure *u*, and the resultant horizontal seismic force *kW*, and is recorded as *σ*_1_; the other part comes from the inter-column forces, and is recorded as *σ*_2_. Equation (17) can be simplified as

18 $$\sigma =\sigma 1+\sigma 2$$

where

19 $${\sigma }_{1}=\frac{(W+P)\cos \,\theta r(\sin \,\beta +\,\cos \,\beta )-kW\,\sin \,\theta r}{A\,\cos \,\theta \,\cos \,\theta r(\sin \,\beta +\,\cos \,\beta )+A\,\sin \,\theta \,\sin \,\theta r(\sin \,\alpha +\,\cos \,\alpha )}-u$$

20 $${\sigma }_{2}=\frac{(\Delta V-\Delta R)\cos \,\theta r(\sin \,\beta +\,\cos \,\beta )-(\Delta T-\Delta H+\Delta F-\Delta E)\sin \,\theta r}{A\,\cos \,\theta \,\cos \,\theta r(\sin \,\beta +\,\cos \,\beta )+A\,\sin \,\theta \,\sin \,\theta r(\sin \,\alpha +\,\cos \,\alpha )}$$

If the slip surface is known, the parameters in Eq. (19) are known; therefore, ^*σ*^_1_ belongs to the known function. Since the distribution of the inter-column forces cannot be accurately determined, *σ*_2_ belongs to the unknown function.

In our work^18^, the following conclusions can be drawn concerning the normal distribution of stresses on the slip surface: (1) in the composition of the normal slip surface stress distribution, the ratio of the weight, external force, the pore water pressure, and the resultant horizontal seismic force (*σ*_1_) to the normal stress (*σ*) is high, while the ratio of the inter-column forces (*σ*_2_) to the normal stress (*σ*) is low; and (2) the normal stresses *σ*, *σ*_1_ and *σ*_2_ are continuous and smooth curves.

## Construction of the Normal Stress Distribution *σ*(*x*′, *y*′)

**Figure 5:**
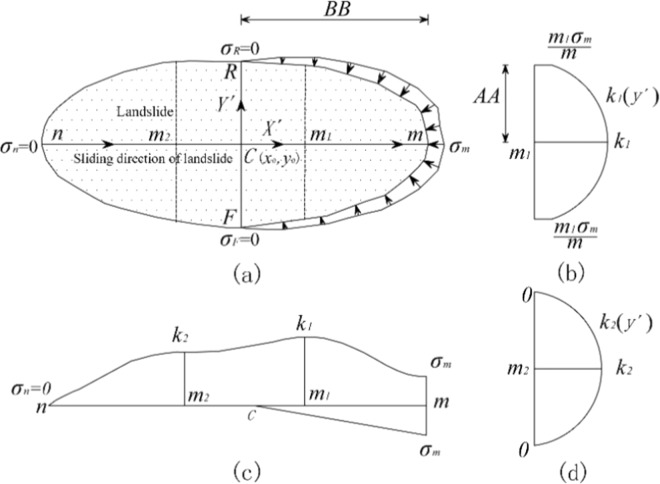
Construction of the normal stress distribution on the slip surface.

In Fig. 5(a), point *C* is the centroid of the landslide. The direction of the long axis, indicated by *nm*, is the sliding direction of the landslide. Points *n* and *m* are the vertex point and the lowest point of the landslide area, respectively. The direction of the shorter axis, indicated by *FR*, is perpendicular to the sliding direction of the landslide. *AA* and *BB* show the size feature of the landslide. By taking two points (*x* = *m*_1_ and *x* = *m*_2_) in the direction of the *X*′-axis, the total normal stresses can be determined, as shown in Fig. 5(c). These two points are chosen as follows:

21 $${m}_{1}=m+1/3(n-m)$$

22 $${m}_{2}=m+2/3(n-m)$$

### Normal stress distribution *σ*(*x*′) along the sliding direction of the landslide

According to Eq. (18), the normal stress distribution along the sliding direction of the landslide can be obtained as follows:

23 $$\sigma (x{\prime} )=\sigma 1(x{\prime} )+\sigma 2(x{\prime} )$$

where *σ*(*x*′) represents the normal stress distribution acting on the slip surface along the sliding direction of the landslide. *σ*_1_(*x*′) represents the component of the weight, external force, and the resultant horizontal seismic force in *σ*(*x*′), which can be determined by the formula in Eq. (19). *σ*_2_(*x*′) represents the component of the inter-column forces in *σ*(*x*′)^.^

If the slip surface is known, *σ*_1_(*x*′) is a known function, and *σ*_2_(*x*′) is an unknown function. The unknown function can be considered to approximate the expression of *σ*_2_(*x*′) using an approximation function. Since *σ*_2_(*x*′) accounts for a small portion of *σ*(*x*′), the approximation function has little effect on the value of *σ*(*x*′), making *σ*(*x*′) close to the actual normal stress distribution.

Assuming that the approximation function is *β*(*x*′), the normal stress distribution *σ*(*x*′) along the sliding direction of the landslide can be expressed as follows:

24 $$\sigma (x{\prime} )={\sigma }_{1}(x{\prime} )+\beta (x{\prime} )$$

Based on the research of Zhu^9^ and Yu^18^, the approximation function *β*(*x*′) is assumed to be a Lagrange polynomial of degree 3; that is,

25 $$\beta (x{\prime} )={k}_{1}{g}_{1}(x{\prime} )+{k}_{2}{g}_{2}(x{\prime} )+{g}_{3}(x{\prime} )$$

where

26 $${g}_{1}(x{\prime} )=\frac{(x{\prime} -m)(x{\prime} -n)(x{\prime} -{m}_{2})}{({m}_{1}-m)({m}_{1}-n)({m}_{1}-{m}_{2})}$$

27 $${g}_{2}(x{\prime} )=\frac{(x{\prime} -m)(x{\prime} -n)(x{\prime} -{m}_{1})}{({m}_{2}-m)({m}_{2}-n)({m}_{2}-{m}_{1})}$$

28 $${g}_{3}(x{\prime} )={\beta }_{m}\frac{(x{\prime} -n)(x{\prime} -{m}_{1})(x{\prime} -{m}_{2})}{(m-n)(m-{m}_{1})(m-{m}_{2})}+{\beta }_{n}\frac{(x{\prime} -n)(x{\prime} -{m}_{1})(x{\prime} -{m}_{2})}{(n-m)(n-{m}_{1})(n-{m}_{2})}$$

where *k*_1_ and *k*_2_ are dimensionless variables; *β*_*m*_ and *β*_*n*_ are the normal stresses acting on the lowest point and the vertex point of the slip surface, respectively.

Since the normal stress at the boundary of the upper half of the landslide is very low, it can reasonably be assumed that the normal stresses at the boundary of the upper half of the landslide are zero^19^ and that *β*_*n*_ = 0. In addition, the normal stresses at the boundary of the bottom half of the landslide are expressed as *σ*_*m*_*x*′/*m*, as shown in Fig. 5(b). *β*_*m*_ is calculated as follows:

29 $${\beta }_{m}=\sigma (m)-{\sigma }_{1}(m)$$

### Normal stress distribution *σ*(*y*′) perpendicular to the sliding direction of the landslide

In the direction perpendicular to the sliding direction of the landslide, the normal stress distribution *σ*(*y*′) is approximated by a parabola along the sliding direction of the landslide, and the apex of the parabola falls on *σ*(*x*′)^18^. Since the apex of the parabola falls on *σ*(*x*′) and the normal stress *σ*(*x*′) is close to the actual normal stress distribution, the accuracy of the function *σ*(*y*′) is controlled. *σ*(*y*′) can be described by

30 $${\sigma }_{b}(y{\prime} )={h}_{1}{y{\prime} }^{2}+{h}_{2}y{\prime} +{h}_{3}$$

31 $${\sigma }_{u}(y{\prime} )={\lambda }_{1}{y{\prime} }^{2}+{\lambda }_{2}y{\prime} +{\lambda }_{3}$$

where *σ*_*b*_(*y*′) is the normal stress *σ*(*y*′) acting on the bottom half of the landslide; *σ*_*u*_(*y*′) is the normal stress *σ*(*y*′) acting on the upper half of the landslide; and *h*_1_, *h*_2_, *h*_3_, *λ*_1_*, λ*_2_ and *λ*_3_ are dimensionless variables.

As shown in Fig. 5(b,d), combined with the characteristics of the parabolic equation, the following can be obtained:

32 $$h1={(A{{A}_{x{\prime} }}^{2})}^{-1}\left(\frac{x{\prime} \sigma (m)}{m}-\sigma (x{\prime} )\right),\,{h}_{2}=0,\,{h}_{3}=\sigma (x{\prime} )$$

33 $$\lambda 1=-\,{(A{{A}_{x{\prime} }}^{2})}^{-1}\sigma (x{\prime} ),\,{\lambda }_{2}=0,\,{\lambda }_{3}=\sigma (x{\prime} )$$

where *AA*^*x*^′ is the shorter axis size corresponding to the abscissa.

### Normal stress distribution *σ*(*x*′, *y*′)

After substituting Eqs. (24), (32,33) into Eqs. (30,31), the normal stress distribution *σ*(*x*′, *y*′) can be determined:

34 $$\{\begin{array}{c}{\sigma }_{b}(x{\prime} ,y{\prime} )={(A{{A}_{x{\prime} }}^{2})}^{-1}{y{\prime} }^{2}\left\{\frac{\sigma (m)}{m}x{\prime} -({\sigma }_{1}(x{\prime} )+\beta (x{\prime} ))\right\}+{\sigma }_{1}(x{\prime} )+\beta (x{\prime} )\\ {\sigma }_{u}(x{\prime} ,y{\prime} )=\{1-{(A{{A}_{x{\prime} }}^{2})}^{-1}{y{\prime} }^{2}\}({\sigma }_{1}(x{\prime} )+\beta (x{\prime} ))\end{array}$$

where *σ*_*b*_(*x*′, *y*′) is the normal stress acting on the bottom half of the landslide, and *σ*_*u*_(*x*′, *y*′) is the normal stress acting on the upper half of the landslide.

The normal stress distribution *σ*(*x*′, *y*′) containing three unknowns (*k*_1_, *k*_2_ and *σ*(*m*)) is determined by substituting Eq. (25) into Eq. (34).

## Solving the 3D Safety Factor

The equation set for calculating the 3D slope safety factor is obtained by substituting Eqs. (2) and (34) into Eqs. (3–6). Now, having four available equations and four unknowns (*SF*_3D_, *k*_1_, *k*_2_ and *σ*(*m*)), these equations can be solved simultaneously by the Newton-Raphson method.

First, a set of initial values (*SF*_3D_^0^, *k*_1_^0^, *k*_2_^0^ and *σ*(*m*)^0^) is applied to obtain four nonzero values: Δ*X*′, Δ*Y*′, Δ*Z* and Δ*M*. Next, this process is repeated until the four nonzero values approach zero. The values for *SF*_3D_, *k*_1_, *k*_2_ and *σ*(*m*) in *l* iterations are determined as follows:

35 $$S{{F}_{3D}}^{l+1}=S{{F}_{3D}}^{l}-{L}_{SF3D}/D$$

36 $${{k}_{1}}^{l+1}={{k}_{1}}^{l}-{L}_{k1}/D$$

37 $${{k}_{2}}^{l+1}={{k}_{2}}^{l}-{L}_{k2}/D$$

38 $$\sigma {(m)}^{l+1}=\sigma {(m)}^{l}-{L}_{\sigma (m)}/D$$

where

39 $$\begin{array}{c}D=|\begin{array}{c}\frac{\partial X{\prime} }{\partial S{F}_{3D}}\,\frac{\partial X{\prime} }{\partial {k}_{1}}\,\frac{\partial X{\prime} }{\partial {k}_{2}}\,\frac{\partial X{\prime} }{\partial \sigma (m)}\\ \frac{\partial Y{\prime} }{\partial S{F}_{3D}}\,\frac{\partial Y{\prime} }{\partial {k}_{1}}\,\frac{\partial Y{\prime} }{\partial {k}_{2}}\,\frac{\partial Y{\prime} }{\partial \sigma (m)}\\ \frac{\partial Z}{\partial S{F}_{3D}}\,\frac{\partial Z}{\partial {k}_{1}}\,\frac{\partial Z}{\partial {k}_{2}}\,\frac{\partial Z}{\partial \sigma (m)}\\ \frac{\partial M}{\partial S{F}_{3D}}\,\frac{\partial M}{\partial {k}_{1}}\,\frac{\partial M}{\partial {k}_{2}}\,\frac{\partial M}{\partial \sigma (m)}\end{array}|,\,LS{F}_{3D}=|\begin{array}{c}\Delta X{\prime} \,\frac{\partial X{\prime} }{\partial {k}_{1}}\,\frac{\partial X{\prime} }{\partial {k}_{2}}\,\frac{\partial X{\prime} }{\partial \sigma (m)}\\ \Delta Y{\prime} \,\frac{\partial Y{\prime} }{\partial {k}_{1}}\,\frac{\partial Y{\prime} }{\partial {k}_{2}}\,\frac{\partial Y{\prime} }{\partial \sigma (m)}\\ \Delta Z\,\frac{\partial Z}{\partial {k}_{1}}\,\frac{\partial Z}{\partial {k}_{2}}\,\frac{\partial Z}{\partial \sigma (m)}\\ \Delta M\,\frac{\partial M}{\partial {k}_{1}}\,\frac{\partial M}{\partial {k}_{2}}\,\frac{\partial M}{\partial \sigma (m)}\end{array}|,\\ {L}_{k1}=|\begin{array}{c}\frac{\partial X{\prime} }{\partial S{F}_{3D}}\,\Delta X{\prime} \,\frac{\partial X{\prime} }{\partial {k}_{2}}\,\frac{\partial X{\prime} }{\partial \sigma (m)}\\ \frac{\partial Y{\prime} }{\partial S{F}_{3D}}\,\Delta Y{\prime} \,\frac{\partial Y{\prime} }{\partial {k}_{2}}\,\frac{\partial Y{\prime} }{\partial \sigma (m)}\\ \frac{\partial Z}{\partial S{F}_{3D}}\,\Delta Z\,\frac{\partial Z}{\partial {k}_{2}}\,\frac{\partial Z}{\partial \sigma (m)}\\ \frac{\partial M}{\partial S{F}_{3D}}\,\Delta M\,\frac{\partial M}{\partial {k}_{2}}\,\frac{\partial M}{\partial \sigma (m)}\end{array}|,\,Lk2=|\begin{array}{c}\frac{\partial X{\prime} }{\partial S{F}_{3D}}\,\frac{\partial X{\prime} }{\partial {k}_{1}}\,\Delta X{\prime} \,\frac{\partial X{\prime} }{\partial \sigma (m)}\\ \frac{\partial Y{\prime} }{\partial S{F}_{3D}}\,\frac{\partial Y{\prime} }{\partial {k}_{1}}\,\Delta Y{\prime} \,\frac{\partial Y{\prime} }{\partial \sigma (m)}\\ \frac{\partial Z}{\partial S{F}_{3D}}\,\frac{\partial Z}{\partial {k}_{1}}\,\Delta Z\,\frac{\partial Z}{\partial \sigma (m)}\\ \frac{\partial M}{\partial S{F}_{3D}}\,\frac{\partial M}{\partial {k}_{1}}\,\Delta M\,\frac{\partial M}{\partial \sigma (m)}\end{array}|,\\ {L}_{\sigma (m)}=|\begin{array}{c}\frac{\partial X{\prime} }{\partial S{F}_{3D}}\,\frac{\partial X{\prime} }{\partial {k}_{1}}\,\frac{\partial X{\prime} }{\partial {k}_{2}}\,\Delta X{\prime} \\ \frac{\partial Y{\prime} }{\partial S{F}_{3D}}\,\frac{\partial Y{\prime} }{\partial k1}\,\frac{\partial Y{\prime} }{\partial {k}_{2}}\,\Delta Y{\prime} \\ \frac{\partial Z}{\partial S{F}_{3D}}\,\frac{\partial Z}{\partial k1}\,\frac{\partial Z}{\partial {k}_{2}}\,\Delta Z\\ \frac{\partial M}{\partial S{F}_{3D}}\,\frac{\partial M}{\partial {k}_{1}}\,\frac{\partial M}{\partial {k}_{2}}\,\Delta M\end{array}|\end{array}$$

In an iterative calculation, the final convergence condition is:

40 $$\begin{array}{c}|{\varepsilon }_{1}|=(S{{F}_{3D}}^{l+1}-S{{F}_{3D}}^{l})/S{{F}_{3D}}^{l} < 0.001\\ |{\varepsilon }_{2}|=({{k}_{1}}^{l+1}-{{k}_{1}}^{l})/{{k}_{1}}^{l} < 0.001\\ |{\varepsilon }_{3}|=({{k}_{2}}^{l+1}-{{k}_{2}}^{l})/{{k}_{2}}^{l} < 0.001\\ |{\varepsilon }_{4}|=(\sigma {(m)}^{l+1}-\sigma {(m)}^{l})/\sigma {(m)}^{l} < 0.001\end{array}\}$$

## Computational Implementation

**Figure 6:**
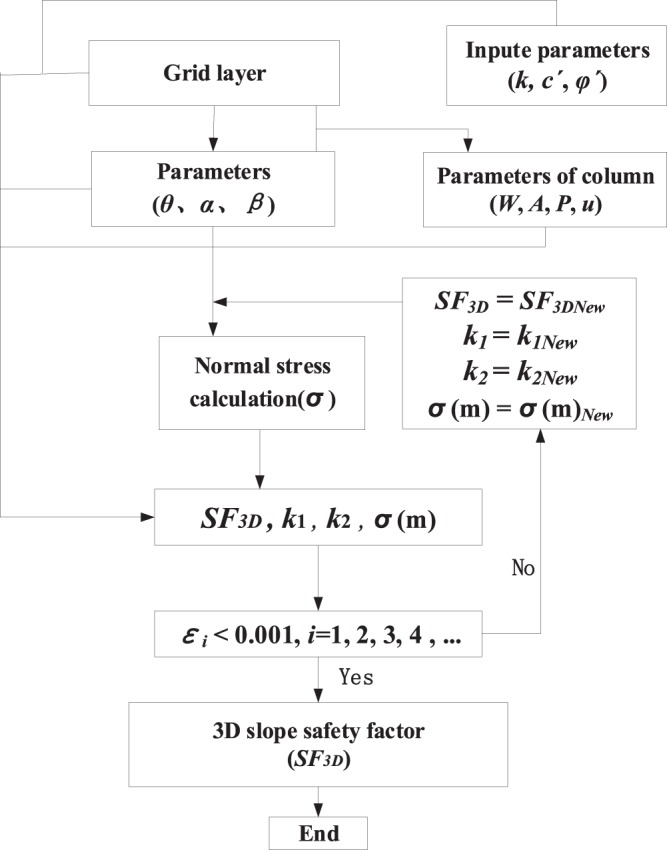
Computational process for 3D slope safety factor.

An extension module for slope stability evaluation has been developed in GIS, and Fig. 6 illustrates the computational process.

## Case Studies

### Case 1: a homogeneous slope

**Figure 7:**
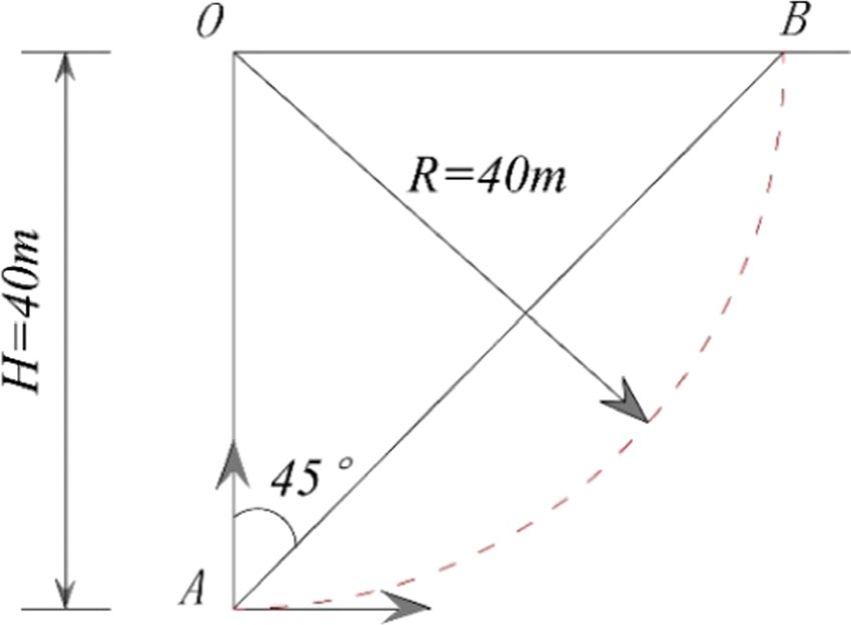
Case 1.

Figure 7 shows a homogeneous slope, with slope angle *β* = 45°, OA = OB, and slope height *H* = 40 m. The unit weight is γ = 22 kN/m³, the effective friction angle is *φ*′ = 30°, and the effective cohesion is *c*′ = 30 kPa. Point *O* is the center of the sliding surface. The slip surface of the case is known. The sizes of grid unit can be set arbitrarily.

The calculation results are shown in Table 1, and the following conclusions can be drawn. 1) The 3D safety factors using the proposed method are similar to those calculated by the 3D M-P method^7^ and Xie’s three GIS-based models^14–16^, i.e., the 3D Hovland model, the 3D Bishop model and the 3D Janbu model. The feasibility of the proposed method is illustrated. 2) Except for the proposed method, the above four methods calculate the safety factor by assuming the inter-column forces, so the obtained safety factor is relatively small relative to the actual value, and the 3D M-P method is closest to the actual value^7^. The result of the proposed method is close to the 3D M-P method, and is 2.7% to 4.5% larger than the 3D M-P method, which is consistent with the pattern that the result value of the 3D M-P method is relatively small relative to the actual value. This result provides more proof that assuming the normal distribution of stress acting on the slip surface of the proposed method is close to the actual normal distribution of stresses.

**Table 1:** The 3D safety factor for Case 1.

Size of grid unit	Hovland 3D	Bishop 3D	Janbu 3D	M-P 3D	Current method
0.5 m	1.138	1.312	1.224	1.368	1.418
0.2 m	1.225	1.369	1.287	1.418	1.463
0.1 m	1.260	1.387	1.317	1.437	1.477

### Case 2: slope with a weak layer

**Figure 8:**
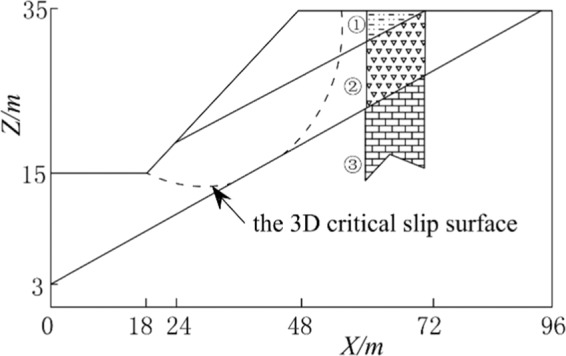
Case 2.

Figure 8 shows a slope with a weak layer sandwiched between two hard, rigid strata. In the soil, the effective cohesion of each of the three layers is 29.4 kPa, 9.8 kPa and 294 kPa; the respective effective friction angles for the three layers are 12°, 5° and 40°; and the unit weight of the three layers is 18.82 kN/m³. The size of the grid element is 0.5 m. In this case, Using a 3D critical slip surface, the 3D safety factor is calculated.

The calculation results are shown in Table 2. In Xie’s research^20^, a minimum 3D safety factor of 0.463 was determined after 100 iterations of a Monte Carlo simulation. Using the 3D M-P approach based on the 3D critical slip surface, a minimum 3D safety factor of 0.496 has been calculated. In this paper, using our proposed method based on the 3D critical slip surface, a higher minimum 3D safety factor of 0.513 was calculated. The outcome of this analysis is near the outcome of the 3D M-P test, and is 3.3% larger than that of the 3D M-P method. This case can draw conclusions similar to those from Case 1.

**Table 2:** The 3D safety factor for Case 2.

Xie’s research	3D M-P	Current method
0.463	0.496	0.513

### Case 3: slope with a discontinuous layer

**Figure 9:**
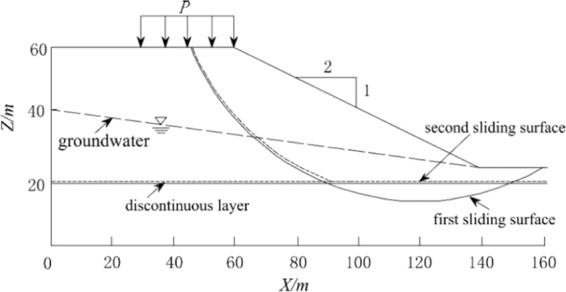
Case 3.

This case shows a slope with a discontinuous layer (Fig. 9). The effective friction angle is 20°, the unit weight is 18.84 kN/m³, and the effective cohesion is 28.7 kPa. The angle of effective friction and the effective cohesion of the discontinuous layer are 20° and 0 kPa, respectively. The slope is considered to have two possible sliding surfaces: the first sliding surface is a cyclic peripheral surface, and the second sliding surface is a nonperfect failure surface. The size of the grid unit is 0.5 m.

The results are presented in Table 3. The calculation result of the current method is the largest and is close to that of the M-P method. This case can draw conclusions similar to those from Case 1 and Case 2. The feasibility of the method is illustrated. The 3D safety factor for the first sliding surface is 2.328. The 3D safety factor for the second sliding surface in the cases with and without groundwater is 1.786 and 1.678, respectively. Moreover, this case considers the calculation of multiple combinations of groundwater, top vertical loads (with load width 5 m, length 10 m) and resultant horizontal forces due to earthquakes. According to Table 2, the impact of an earthquake on the safety factor is high, and the occurrence of an earthquake produces results that are among the most unfavorable combinations of factors. Considering the combinations of earthquake and top vertical loads (160 kN/m³), the safety factor is below the maximum design allowance (1.20). The case study results indicate that this module can readily be used to calculate various loads in slope design combinations.

**Table 3:** The safety factor for Case 3.

	Combination	Hovland 3D	Modified Hovland 3D	Bishop 3D	Janbu 3D	M-P 3D	Current method
The first sliding surface		2.091	2.302	2.282	2.043	2.312	2.328
The second sliding surface	1. NG	1.568	1.672	1.711	1.609	1.764	1.786
	2. GG	1.485	1.569	1.620	1.536	1.671	1.678
	3. GG + RQ	1.298	1.319	/	1.359	1.495	1.499
	4. GG + VD(5)	1.478	1.564	1.613	1.527	1.657	1.661
	5. GG + VD(10)	1.476	1.558	1.606	1.517	1.652	1.657
	6. GG + VD(20)	1.456	1.547	1.592	1.499	1.642	1.647
	7. GG + VD(40)	1.427	1.526	1.565	1.463	1.613	1.617
	8. GG + VD(80)	1.374	1.485	1.515	1.399	1.557	1.561
	9. GG + VD(160)	1.281	1.412	1.427	1.290	1.479	1.481
	10. (9) + RQ	1.141	1.209	/	1.166	1.217	1.221

Note: (1) NG represents no groundwater, and GG represents groundwater.

(2) RQ represents the resultant horizontal force due to an earthquake, *k* = 0.05.

(3) VD(5) represents vertical loads, and *p* = 5 kN**/**m³.

## Discussion

### Discussion on the advantages of GIS-based slope stability analysis method

GIS provides a common platform for multi-source data, and all slope-related data can be converted to GIS raster datasets. Combined with the advantages of GIS in processing complex spatial data, the limit equilibrium method can be easily extended to three dimensions. By adding a professional model to the GIS, a three-dimensional slope stability analysis model based on grid column unit can be established to analyze the stability of the three-dimensional slope. The model has the advantages of simplicity and simple programming.

### Discussion on the advantages of the approximation function

In this paper, the normal stress distribution *σ*(*x*′) along the sliding direction of the landslide is composed of *σ*_1_(*x*′) and *β*(*x*′), where *σ*_1_(*x*′) is a known function. Since the approximation function *β*(*x*′) has a small proportion in *σ*(*x*′), the approximation function has a small influence on the result value of *σ*(*x*′). For the normal stress distribution *σ*(*y*′) perpendicular to the sliding direction of the landslide, since the apex of the parabola falls on *σ*(*x*′), the accuracy of *σ*(*x*′) controls the accuracy of the function *σ*(*y*′). In general, the method proposed in this paper is different from the traditional method in that it does not need to ignore the effect of the inter-column forces, and the assumed normal stress distribution function has less influence on the result value, so the resulting value calculated by using the method proposed in this paper is closer to the actual value.

### Discussion of the sliding direction of the landslide

The sliding direction of the landslide refers to the direction in which the slider body begins to slide. In the limit equilibrium method of this paper, it is necessary to establish the limit equilibrium equation and the assumed normal stress distribution function of the slip surface along the sliding direction of the landslide, as in Eqs. (3) and (25). However, before the landslide slides, the sliding direction of the landslide is unknown, so this paper assumes that the sliding direction of the landslide is the main dip direction of all the grid columns. For each grid column unit of the landslide, the dip direction of the slip surface is different, and the main dip direction is the most frequent value of the dip direction of all grid columns in the range of the sliding body.

## Conclusions

This study proposes a method for analyzing stability of the 3D slope based on GIS which assumes a normal stress distribution that works on the slip surface. The 3D limit equilibrium equations to solve the 3D safety factor are derived from the grid column unit model, and the spatial representation of the calculation parameters is given in GIS.

An approximation function is proposed by analyzing the composition of normal stress distribution to approximate the actual normal stress distribution that operates on the slip surface. Compared with traditional methods, the resulting value calculated by using the approximation function is closer to the actual value. This approximation function provides a theoretical basis for the establishment of a GIS-based slope stability analysis method based on the assumption of normal slip surface stress distribution.

To assess the stability of the slope a GIS-based extension module is built. In this module, the slope data is derived directly from the GIS dataset, therefore, the module has the advantages of a uniform data format and simple data preparation process. The module can be used for multiple stratigraphic slopes as well as stability calculations under a variety of combined loads. The accuracy and feasibility of the module are verified by three typical cases.

## Data Availability

All the data in this article is available.

## References

[1] Liu SY, Shao LT, Li HJ (2015). Slope stability analysis using the limit equilibrium method and two finite element methods. Computers and Geotechnics.

[2] Cheng YM, Lansivaara T, Wei WB (2017). Two-dimensional slope stability analysis by limit equilibrium and strength reduction methods. Computers and Geotechnics.

[3] Zhou XP, Cheng H (2013). Analysis of stability of three-dimensional slopes using the rigorous limit equilibrium method. Engineering Geology.

[4] Hovland JH (1979). Three-dimensional slope stability analysis method. Journal of Geotechnical and Geoenvironmental Engineering.

[5] Hungr O (1987). An extension of Bishop’s simplified method of slope stability analysis to three dimensions. Géotechnique.

[6] Boutrup E, Lovell CW (1980). Searching techniques in slope stability analysis. Engineering Geology.

[7] Deng DP, Zhao LH, Li L (2016). Limit equilibrium method for slope stability based on assumed stress on slip surface. Journal of Central South University.

[8] Bell JM (1968). General slope stability analysis. Journal of the Soil Mechanics and Foundations Division.

[9] Zhu DY, Lee CF (2002). Explicit limit equilibrium solution for slope stability. International Journal for Numerical & Analytical Methods in Geomechanics.

[10] Zhu, D. Y., Ding, X. L., Du, J. H. & Deng, J. H. Computation of 3D safety factor of asymmetric and rotational slopes. *Chinese Journal of Rock Mechanics and Engineering* **29**, 1236–1239 (in Chinese) (2007).

[11] Zhu, D. Y. & Qian, Q. H. Rigorous and quasi-rigorous limit equilibrium solutions of 3D slope stability and application to engineering. *Chinese Journal of Rock Mechanics and Engineering* **26**, 1513–1528 (in Chinese) (2007).

[12] Zheng H, Tham LG (2010). Improved Bell’s method for the stability analysis of slopes. International Journal for Numerical & Analytical Methods in Geomechanics.

[13] Carrara A, Pike RJ (2008). GIS technology and models for assessing landslide hazard and risk. Geomorphology.

[14] Xie M, Esaki T, Zhou G, Yasuhiro M (2003). Geographic information systems-based three-dimensional critical slope stability analysis and landslide hazard assessment. Journal of Geotechnical and Geoenvironmental Engineering.

[15] Xie M, Esaki T, Qiu C, Wang CX (2006). Geographical information system-based computational implementation and application of spatial three-dimensional slope stability analysis. Computers and Geotechnics.

[16] Xie M, Esaki T, Cai M (2006). GIS-based implementation of three-dimensional limit equilibrium approach of slope stability. Journal of Geotechnical and Geoenvironmental Engineering.

[17] Mergili M, Marchesini I, Rossi M, Fausto G, Fellin WF (2014). Spatially distributed three-dimensional slope stability modelling in a raster GIS. Geomorphology.

[18] Yu, G., Xie, M. W., Sun, Z. H. & Liu, P. Approximation function structure of sliding surface normal stress distribution of three-dimensional symmetrical slope based on GIS. *Rock and Soil Mecha*nics **40**, 2332–2340 (in Chinese) (2019).

[19] Leshchinsky D (1990). Slope stability analysis: generalized approach. Journal of geotechnical engineering.

[20] Xie, M. W. & Cai, M. F. Theory and practice of information slope engineering (*Science Press*) 40–41 (Beijing, 2005).

